# Electromagnetic field and TGF-β enhance the compensatory plasticity after sensory nerve injury in cockroach *Periplaneta americana*

**DOI:** 10.1038/s41598-021-85341-z

**Published:** 2021-03-22

**Authors:** Milena Jankowska, Angelika Klimek, Chiara Valsecchi, Maria Stankiewicz, Joanna Wyszkowska, Justyna Rogalska

**Affiliations:** 1 Department of Animal Physiology and Neurobiology, Faculty of Biological and Veterinary Sciences, Nicolaus Copernicus University, Lwowska 1, 87-100 Toruń, Poland; 2 Federal University of Pampa, Campus Alegrete, Alegrete, RS Brazil

**Keywords:** Biological techniques, Biophysics, Neuroscience, Physiology, Environmental sciences, Neurology

## Abstract

Recovery of function after sensory nerves injury involves compensatory plasticity, which can be observed in invertebrates. The aim of the study was the evaluation of compensatory plasticity in the cockroach (*Periplaneta americana)* nervous system after the sensory nerve injury and assessment of the effect of electromagnetic field exposure (EMF, 50 Hz, 7 mT) and TGF-β on this process. The bioelectrical activities of nerves (pre-and post-synaptic parts of the sensory path) were recorded under wind stimulation of the *cerci* before and after right *cercus* ablation and in insects exposed to EMF and treated with TGF-β. Ablation of the right *cercus* caused an increase of activity of the left presynaptic part of the sensory path. Exposure to EMF and TGF-β induced an increase of activity in both parts of the sensory path. This suggests strengthening effects of EMF and TGF-β on the insect ability to recognize stimuli after one *cercus* ablation. Data from locomotor tests proved electrophysiological results. The takeover of the function of one *cercus* by the second one proves the existence of compensatory plasticity in the cockroach escape system, which makes it a good model for studying compensatory plasticity. We recommend further research on EMF as a useful factor in neurorehabilitation.

## Introduction

Injuries in the nervous system caused by acute trauma, neurodegenerative diseases or even old age are hard to reverse and represent an enormous challenge for modern medicine. Function recovery after injury involves two main processes: (1) compensatory plasticity—reorganization of neuronal circuits that have been affected by the laceration or (2) regenerative processes in lesioned axons^1,2^.

Compensatory plasticity and functional recovery after injury of sensory-motor systems have already been reported in insects^3,4^. Many neurons are “re-sculptured” during the normal course of insect post-embryonic development, especially during metamorphosis^5,6^. Moreover, the surgical elimination of an input source into the central nervous area triggers the plasticity processes: the sprouting of axons, the formation of new connections, the formation of a bypass to enter the central nervous system through other routes^7–9^. The simplicity of the insect nervous system, together with the opportunity to study already identified neurons or neuronal circuits make insects an advantageous model system for neurorehabilitation research.

One of the most well-established model organisms in neurobiological research is the American Cockroach (*Periplaneta americana*)^10-12^. The escape system of *Periplaneta americana* could be extremely valuable for research on nerve function restoration. The sensory part of the cockroach escape system contains wind-receptive hairs located on two posterior abdominal appendages, the *cerci*. Axons of wind-receptive sensory neurons (forming cercal nerve) are connected with giant interneurons (GI) in the terminal abdominal ganglion (TAG). The information received by the sensory hairs is transported to the leg motor neurons via these interneurons^13,14^. Cockroaches can distinguish the wind currents from the left and right sides and turn away in directions opposite to the wind source^13^.

Initial steps of adaptive mechanisms underlying the restoration of lost function always occur first at the molecular level and eventually lead to structural alternations. The prominent example of a multifunctional agent driving plasticity is the Transforming Growth Factor-β (TGF-β), which modulates cellular survival and growth in vertebrates. In invertebrates administration of TGF-β, following injuries to the nervous system, regulates the functions of neurons and glial cells, thus, mediating the plasticity processes^15,16^.

The efficiency of compensatory plasticity is also modulated by external stimuli. Exposure to electromagnetic field (EMF) is one of the factors potentially useful in improving nerve system functions, increasing the ability to neuronal plasticity and nerve regeneration. Exposure to EMF induces neuronal differentiation and potentiates synaptic transmission and plasticity^17–19^.

This work aims to verify whether the exposure to EMF (50 Hz, 7 mT) will enhance the plasticity in the cockroach nervous system and as a result, restore the cockroach's ability to respond to air puff stimuli after the removal of one *cercus*.

## Results

### In-vitro electrophysiology

#### Bioelectrical activity of the Periplaneta americana escape system nerves

**Figure 1:**
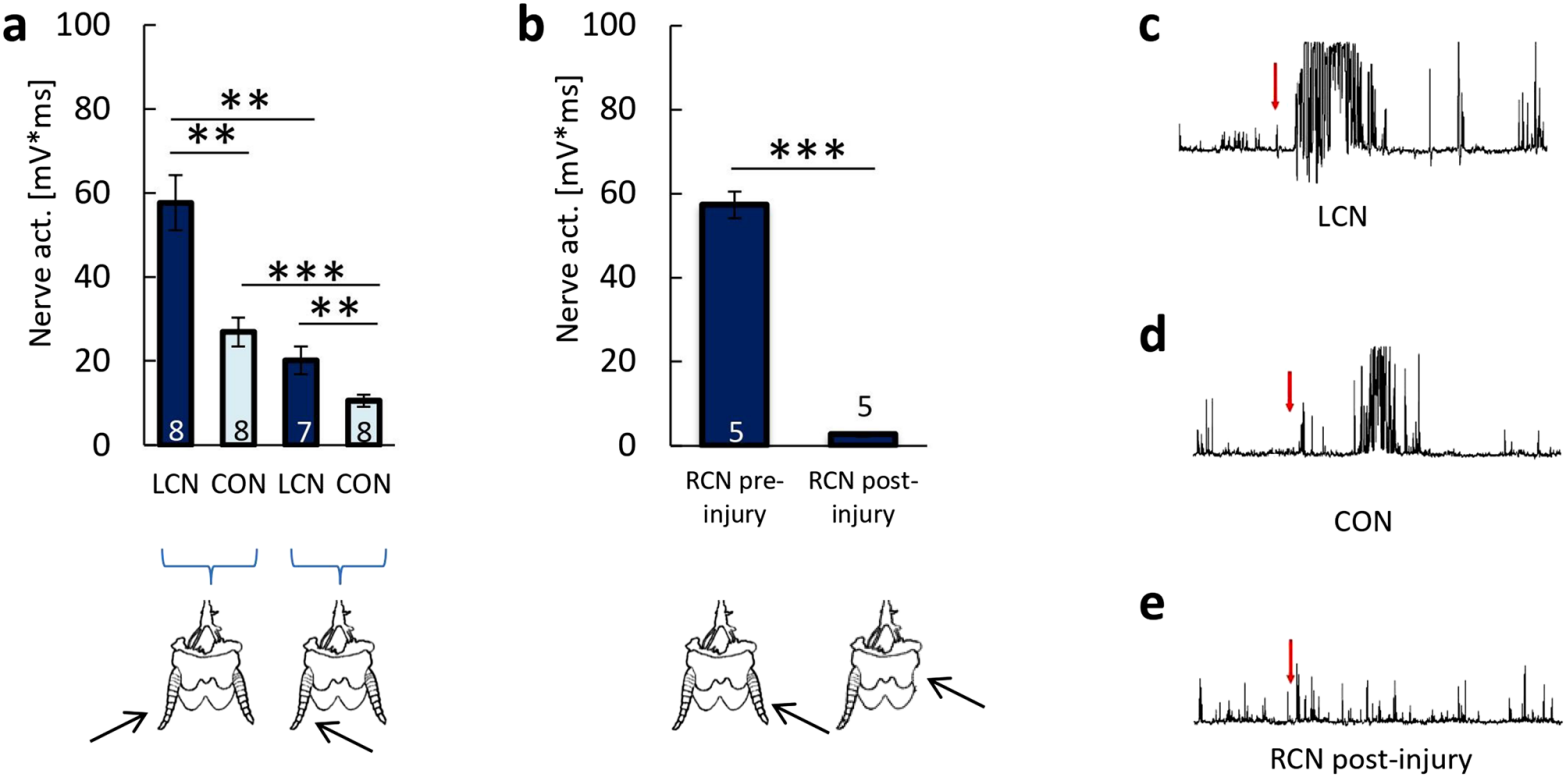
The neuronal activity of the escape system of American Cockroach (*Periplaneta americana*). (**a**) The level of nerve activity of the left part of the escape system. Recordings were performed on the left cercal nerve (LCN) and connective nerve (CON), after stimulation of the left *cercus* (LC) from the left and right side (indicated by arrows). The data were expressed as mean values ± SE; sample size n is indicated on the bars; the statistically significant differences: **p < 0.01, ***p < 0.001. (**b**) The level of activity of the right cercal nerve (RCN): before the injury—“RCN pre-injury” (recorded in NI-Ctr group) and 24 h after the injury “RCN post-injury” (recorded in I-Ctr group). The data are expressed as mean values ± SE, sample size n is indicated on the bars, the statistically significant differences: ***p < 0.001. (**c**–**e**) Original recordings: from (**c**) LCN and (**d**) CON after LC stimulation from the left side and (**e**) RCN after RC ablation.

In the first set of experiments, the activity of nerves in NI-Ctr group was evaluated. The activity of the left cercal nerve (LCN) under the left *cercus* (LC) stimulation from the left side was 38.95% higher than LCN activity when LC was stimulated from the right side (Fig. 1a) (p = 0.0065). Right *cercus* (RC) responded better to stimulations from the right side, and activity of right cercal nerve (RCN) when RC was stimulated from right side was similar to the activity of LCN, when LC was stimulated from the left (Fig. 1a,b). These results showed that LC responds the most to left side stimulation, whereas the highest response of RC occurs under stimulation from the right side of the body.

When LC was stimulated from the left, the activity of the connective nerve (CON) was 53.37% lower than the activity of LCN after stimulation from the same direction (p = 0.01; Fig. 1a). A similar reduction in CON activity was observed when LC was stimulated from the right: the activity of CON was 52.90% lower than LCN activity after receiving the stimulation from the same side (p = 0.0032). Original representative recordings of LCN and CON activity when LC was stimulated from the left side are presented in Fig. 1c,d, respectively.

#### Changes in the bioelectrical activity of the escape system nerves after ablation of the right cercus

RC ablation resulted in the loss of the ability to perceive stimulation (Fig. 1b,e). Shortly after RC ablation, LCN, and CON activities, regardless of the side of LC stimulation, were not significantly different from the pre-injury values. That indicates that immediately after RC ablation, the perception of the right-sided stimulation was limited, and the enhancement of the response was not observed.

**Figure 2:**
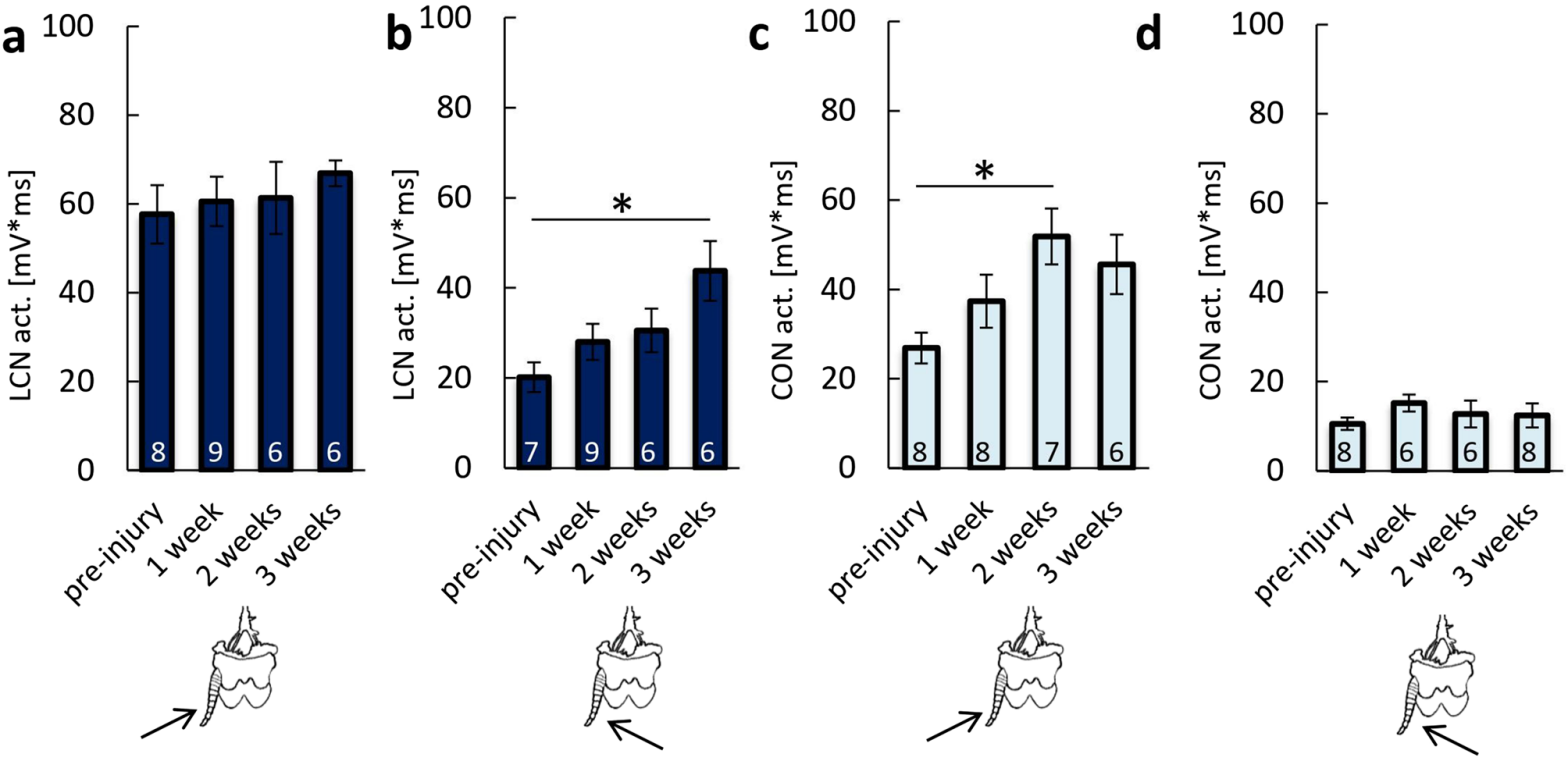
Neuronal activity of the escape system for the I-Ctr group. Pre-injury values were evaluated in the NI-Ctr group. The assessment was conducted for 3 weeks on: (**a**) the left cercal nerve after stimulation of the left *cercus* from the left side; (**b**) the left cercal nerve after stimulation of the left *cercus* from the right side; (**c**) the connective nerve after stimulation of the left *cercus* from the left side; (**d**) the connective nerve after stimulation of left *cercus* from the right side. All data were expressed as mean values ± SE, sample size n is indicated on the bars, the statistically significant differences: *p < 0.05.

In the I-Ctr group, LCN activity after LC stimulation from the left side was constant over a period of 3 weeks (Fig. 2a). The slight increase in nerve activity, observed in the 3rd week of the experiment, was not significant. However, the activity of LCN after stimulation from the right side increased over time (Fig. 2b). After 3 weeks, LCN activity was 94.93% higher than the activity before the injury (p = 0.043). In the I-Ctr group, changes in the activity of CON were also observed. When LC was stimulated from the left, CON activity increased (Fig. 2c); particularly, 2 weeks after the injury, the activity was 92.95% higher than pre-injury (p = 0.036). When LC was stimulated from the right, CON activity remained at the pre-injury level up to the end of the observations (Fig. 2d).

#### Effect of EMF exposure on the changes in the bioelectrical activity of the escape system nerves after ablation of the right cercus

**Figure 3:**
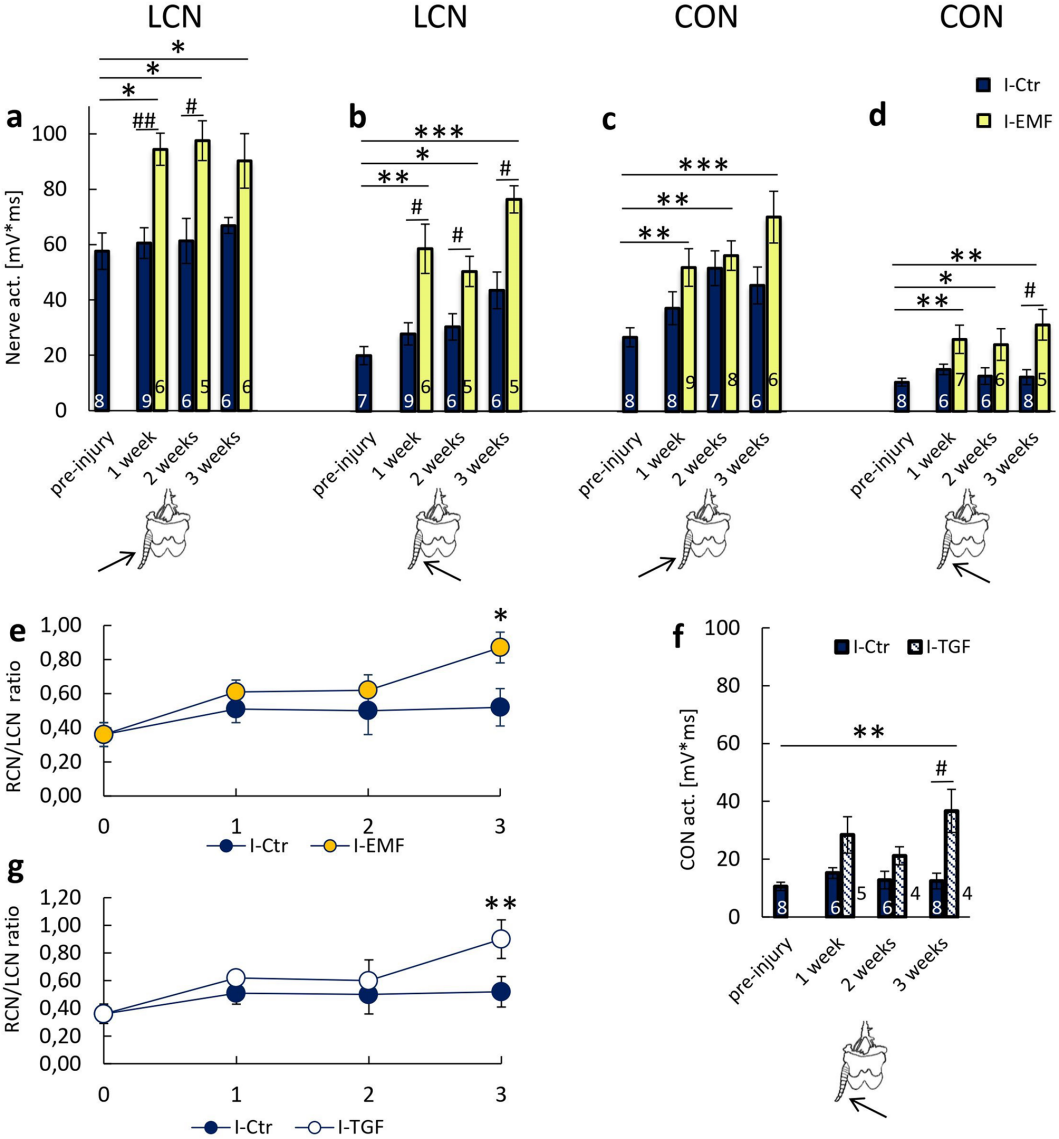
The effect of electromagnetic field exposure on the neuronal activity of cockroach escape system. (**a**–**d**) The neuronal activity in the I-Ctr group (navy bars) and in the I-EMF group (yellow bars) assessed for: (**a**) LCN after stimulation of the left *cercus* from the left side; (**b**) LCN after stimulation of left *cercus* from the right side; (**c**) CON after stimulation of left *cercus* from the left side; and (**d**) CON after stimulation of left *cercus* from the right side. Pre-injury values were evaluated in the NI-Ctr group. (**e**) The ratio of cercal nerve activity after left *cercus* stimulation from the right side in respect to the left side (R/L ratio) for I-Ctr group and I-EMF group. (**f**) CON activity in the I-TGF group (positive control), when the left *cercus* was stimulated from right side. (**g**) Cercal nerve activity ratio after stimulation of *cercus* from the right side in respect to the left side, for I-Ctr group and I-TGF group. All data were expressed as mean values ± SE, sample size n is indicated on the bars. The statistically significant differences in the I-EMF group and the pre-injury values (NI-Ctr group) are marked as *p < 0.05, **p < 0.01, ***p < 0.001, while differences between the I-EMF and the I-Ctr groups are marked as ^#^p < 0.05, ^##^p < 0.01.

LCN and CON activities were evaluated in cockroaches exposed to electromagnetic field (I-EMF group). In this group, LCN activity in response to stimulation of LC from the left side was higher by 55.96%, 59.12%, and 34.89% after 1, 2, and 3 weeks, respectively, than in the I-Ctr animals, (p = 0.005 for 1 week after injury, p = 0.017 after 2 weeks; Fig. 3a). These values were also higher by 63.85%, 69.26%, and 56.57% than the pre-injury values (p = 0.018 after 1 week, p = 0.014 after 2 weeks and 0.042 after 3 weeks).

The increase in LCN activity was also observed when LC was stimulated from the right side (Fig. 3b). LCN activity was higher by 110.05%, 65.62%, and 75.33%, respectively, than the activity in the I-Ctr group at corresponding times (p = 0.012 after 1 week, p = 0.030 after 2 weeks, p = 0.017 after 3 weeks) and was higher by 162.07%, 125.46% and 241.79%, respectively, than the pre-injury LCN activity (p = 0.003 for 1 week, p = 0.034 for 2 weeks and p < 0.001 for 3 weeks).

The EMF induced an increase of CON activity (Fig. 3c,d). When LC was stimulated from the left, the CON activity was only slightly higher than the activity of the nerve found in the I-Ctr group. However, the CON activity for the I-EMF group were higher than the pre-injury nerve activity by 109.85%, and 161.65%, respectively, after 2, and 3 weeks (p = 0.01 for 1 week, p = 0.004 for 2 weeks, p = 0.001 for 3 weeks) (Fig. 3c).

When LC was stimulated from the right side, an increase in CON activity was also observed (Fig. 3d). The values were 71.38%, 89.51% and 152.21% higher than the values in I-Ctr group, after 1, 2 and 3 weeks, respectively (p = 0.023 after 3 weeks), moreover the values were 146.36%, 128.58% and 196.18% higher than CON activity measured before the injury (p = 0.005 for 1 week, p = 0.024 for 2 weeks and p = 0.004 for 3 weeks).

To evaluate whether the perception of the right-sided stimulation was overtaken by the left side of the escape system, the ratio of LCN activity induced by the right sided stimulation in respect to the stimulation from the left side was calculated—R/L ratio (Fig. 3e). For the I-Ctr group, the ratio increased from 0.36 ± 0.07, before injury, to 0.51 ± 0.08 in 1 week after injury, and stayed constant afterwards: in 2 weeks—0.50 ± 0.14 and in 3 weeks—0.52 ± 0.11. In I-EMF group, this ratio increased to 0.61 ± 0.07 in 1 week after injury, to 0.62 ± 0.09 after 2 weeks and to 0.87 ± 0.09 in 3 weeks after injury (p = 0.014). Higher values of the ratio indicate better perception of right-sided stimulation by the left side of the escape system. It should be emphasized that the increase over time of the R/L ratio was only observed for the I-EMF group, and not for the I-Ctr group.

#### Effect of TGF-β on the changes in the bioelectrical activity of escape system nerves after ablation of the right cercus

To verify if the effect of EMF exposure was not accidental or non-specific, TGF-β was used as a positive control (Fig. 3f). The magnitude of the bioelectrical activity of CON was evaluated when LC was stimulated from right side since the EMF exposure affected the most CON activity.

When TGF-β was administrated, CON activity was found higher by 86.74%, 65.93%, and 95.32% in respect to the I-Ctr group, for 1, 2, and 3 weeks, respectively (p = 0.017 for 3 weeks), and the values were found increased by 68.43%, 100.13%, and 246.8% when compared to pre-injury values (p = 0.008 for 3 weeks). CON activity after TGF-β treatment was not significantly different from the activity registered when insects were exposed to EMF (see Fig. 3d).

TGF-β impact on the R/L ratio was also evaluated (Fig. 3g). The level of R/L ratio was equal to 0.62 ± 0.05 in 1 week after injury, 0.60 ± 0.15 after 2 weeks, and 0.90 ± 0.14 after 3 weeks. In the third week of the experiment, the R/L ratio was significantly higher in insects treated with TGF-β in respect to pre-injury (p = 0.009) and did not differ from the ratio evaluated in insects exposed to EMF (see Fig. 3e).

### In-vivo behavioral test

**Figure 4:**
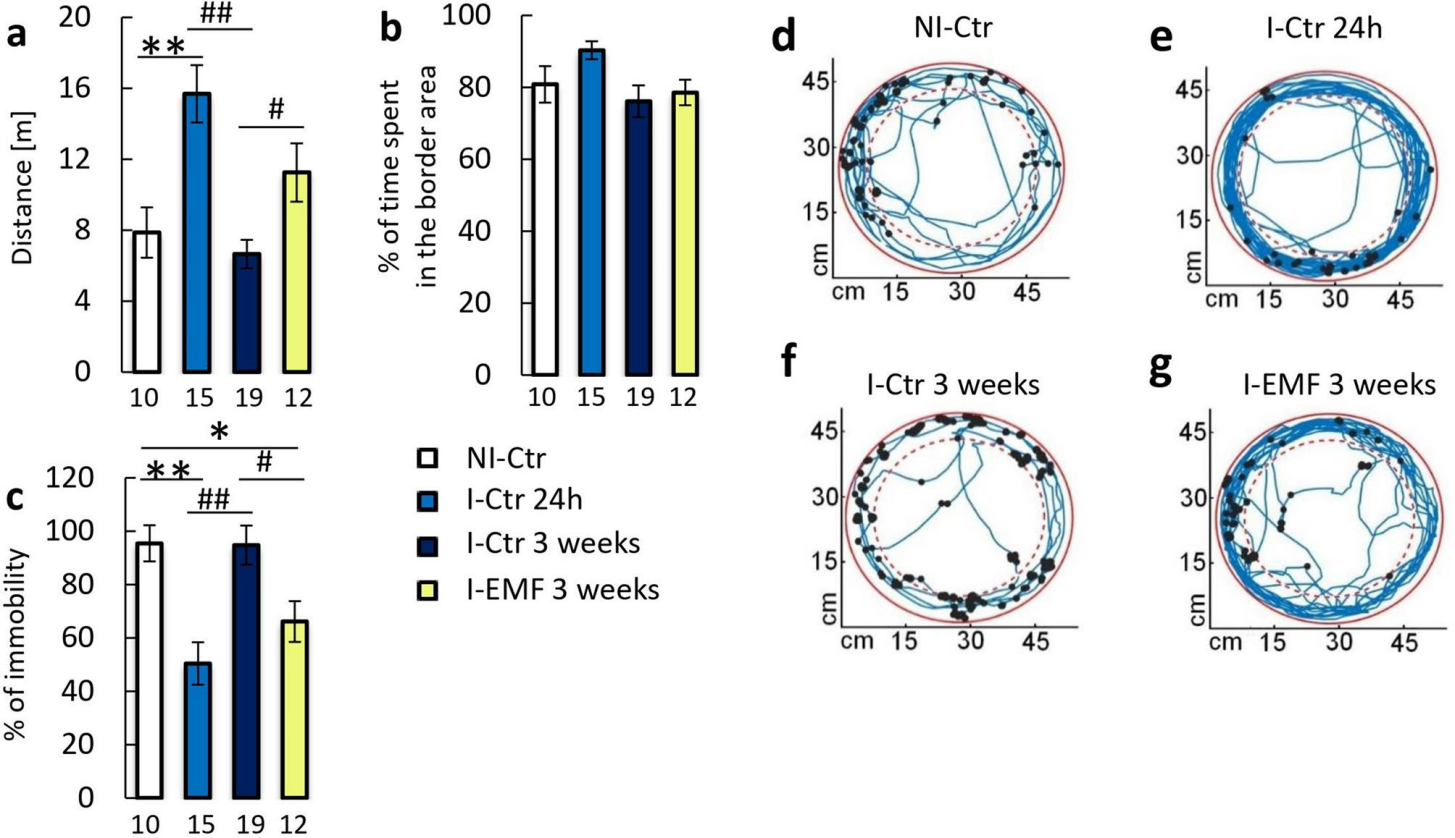
Locomotor activity of the *Periplaneta americana*. (**a**) Distance moved by cockroaches; (**b**) Percentage of time spent at the arena border, defined as 10% of the diameter; (**c**) Percentage of time spent in immobility; (**d**–**g**) Tracker recordings of insect movements in the arena. Insect movement is represented as a blue line, while the immobility events are represented as black dots. Recording are presented for (**d**) not-injured control group; (**e**) injured control group 24 h after the injury, (**f**) injured control group 3 weeks after injury, (**g**) injured, EMF exposed group, after 3 weeks. All data were expressed as mean values ± SE, sample size n is indicated on the bars. The statistically significant differences in the I-EMF group and the pre-injury values (NI-Ctr group) are marked as *p < 0.05, **p < 0.01, while differences between the I-EMF and the I-Ctr groups are marked as ^#^p < 0.05, ^##^p < 0.01. The charts (**d**–**g**) were generated in MATLAB R2020a (https://www.mathworks.com).

The *cerci* are the sensory part of the *Periplaneta americana* escape system. Therefore, depriving the cockroach of these appendices should influence its behavior. We evaluated the locomotor behavior of the cockroach using an animal tracking system (Fig. 4). 24 h after the injury, the I-Ctr insects traveled a distance almost two times higher than before the injury (NI-Ctr) (p = 0.006) and stayed closed to the arena border slightly longer than before the injury (Fig. 4a,b,d,e).

I-Ctr cockroaches deprived of the RC, 3 weeks after the injury, traveled a distance similar to that of NI-Ctr insects. Moreover, they remained close to the arena borders similarly to the NI-Ctr animals (Fig. 4a,b,f). The distance traveled by cockroaches deprived of RC and exposed to EMF for 3 weeks was slightly higher than the distance reached by insects before the injury and significantly higher than that in I-Ctr insects (p = 0.03). However, the I-EMF insects remained at the arena border for a time similar to the NI-Ctr and I-Ctr groups (Fig. 4a,b,g).

The insects deprived of RC were significantly more active 24 h after the injury than the NI-Ctr animals (p = 0.002) (Fig. 4c–e). Three weeks after the injury in the I-Ctr group, the percentage of motionless returned to the pre-injury value (Fig. 4c,f). Surprisingly, in the I-EMF group, the immobility was significantly lower than in the I-Ctr group and NI-Ctr group (p = 0.027 and p = 0.038 respectively; Fig. 4c,g).

## Discussion

After the loss of peripheral sensory parts, the central nervous system adapts to the new situation through compensatory mechanisms, such as neuronal plasticity and/or taking over the lost function by other neurons. Compensatory plasticity is observed among all groups of animals, including humans: i.e., loss of vision or hearing leads to an increase in the performance of part of cortex processing information from other senses^20^. Similarly to vertebrates, crickets with lesioned ear first lose the ability to recognize the sound direction, however, over time, the directionality improves^21^. It is important to mention that the compensatory plasticity in invertebrates can be successfully observed through adult life, and thus, making them a valuable model to examine the mechanisms of compensation.

*Periplaneta americana* escape system has been used in many neurobiological studies, although, for the first time, we have used this model to study the plasticity processes. Each sensory part of this system is designated to detect a stimulation, e.g., wind currents, but also, it can precisely determine the direction from which the stimuli are coming. The information on air movement comes from the combined activity of the filiform hairs covering *cerci*, each of them with a specific location on the appendix and biomechanical features^22,23^. In our experiments, it was shown that the sensitivity of each of the *cercus* is different to air puffs directed from its right or left side. Significantly larger responses were recorded from cercal nerves when *cerci* were stimulated from “external” directions (left *cercus* from the left, right *cercus* from the right) than from “internal” ones.

After right *cercus* (RC) ablation, almost non-activity was detected at right cercal nerve (RCN). Cell bodies with dendrites and axon parts of sensory cells were cut off and the stimuli reception was impossible. Moreover, it is known that axons of the cercal nerve degenerate very rapidly after the removal of the *cercus*^24^. As a result, left-sided stimuli were detected stronger than right-sided stimuli.

RC ablation, shortly after the injury, did not affect left cercal nerve (LCN) activity regardless of the direction of stimulation; activity recorded from connective nerve (CON) also remained the same. However, observations of LCN activity showed that left *cercus* (LC) becomes more and more sensitive to stimulation from the right over time. The stimulation effect from the left side was not changed. This indicates that LC’s ability to recognize stimulation from the right side improved. A significant increase in LCN activity after 3 weeks can result from changes in the receptive field of hair mechanoreceptors, the sensitivity of sensory cells and encoding efficiency. The phenomenon of plasticity following injury is well known in the nervous system of adult organisms and it is referred to as “lesion-induced plasticity”, or more specifically—compensatory plasticity. The term “plasticity” is mainly associated with modifications in the synaptic transmission, but this term is also used for various changes in the nervous system functions in response to external and internal stimuli^25^. The question arises: what are the processes underlying such a modification, manifested by an increase in the activity of LCN?

In the cockroach escape system, the cercal sensory cells are connected with giant interneurons (GI) through ipsilateral and contralateral projections, and all synapses are located in the terminal abdominal ganglion. Each GI plays a particular role in processing information about the stimuli acting on *cerci*^26^. GI axons are the main components of the CON. RC ablation “traumatized” GIs—it means that the reduced stimulation reaching the GIs may be the factor triggering “retrograde reaction” in GIs. The synapse strength regulation by retrograde messengers is well known; postsynaptic neurons secrete different types of molecules that can activate presynaptic receptors to directly regulate neurotransmitter release but may also (through growth factors) modify survival, gene expression, and properties of the presynaptic sensory neurons^27,28^. There are evidences that released retrograde messengers can specifically modulate the function of only the active presynaptic neurons^29^. These mechanisms can be classified as functional, non-synaptic plasticity, which is associated with changes in the intrinsic properties of neurons^30^. The observed increase of activity of the sensory cells of LCN leads to the conclusion that such processes can be recognized as the plasticity at the level of a single neuron. Over time, no changes in CON activity were observed after the right-sided stimulations of LC. This suggests that, even if the sensory neurons become more sensitive to the right-sided stimuli, they did not make new connections to existing structures (GIs). The process of forming the new neuronal connections is not fully understood, although, it is known that axonal growth and formation of new synaptic connections are more likely to occur in more active neurons, which can recognize and uptake the trophic factors^31,32^.

A significant increase in the bioelectrical activity of the CON after LC stimulation from the left side was already observed 2 weeks after RC ablation. Two factors may be responsible for this increase: (1) non-synaptic modification of cercal sensory neuron function and (2) increase of the synapse's strength between sensory neurons and GIs, induced by the influence of “traumatized” GI. It was demonstrated that, when axons of many neurons form synaptic connections with one neuronal or muscle cell, they compete with each other for the limited amount of trophic factors, produced by the neuronal/muscle cell. When synaptic endings of dying axons (from ablated RC) become inactive, the remaining synaptic connections (formed by the left sensory neurons) are strengthened^31^.

We have exposed insects to EMF of 50 Hz and 7 mT. Such parameters are within a range most commonly applied in magnetotherapy^33,34^. EMF exposure significantly increased LCN activity in response to LC stimulation from both sides. In our previous experiments, EMF exposure (50 Hz, 7 mT) induced a reduction in the threshold of response to stimuli^35^. Exposure to EMF modifies the intracellular concentration of Ca^2+^, which results from the higher activity of Ca^2+^ membrane channels and/or additional release of Ca^2+^ from intracellular resources. Changes in calcium-dependent signaling in sensory neurons may be responsible for a significant increase in the responsiveness of LCN to stimulation following EMF exposure^19,36^.

The specific increase of Ca^2+^ and cyclic nucleotides, as well as the presence of neuronal growth factors, determines the direction of axon growth and thus, the location of new synaptic connections. It was shown that, in fibroblast cultures, EMF may affect several membrane receptors for TGF-β1. Moreover, EMF can stimulate the secretion of growth factors such as TGF-β in different mammalian cells^37,38^. In our study, a significant increase of CON activity in insects exposed to EMF was observed. This increase can be a result of new synaptic connections between left cercal sensory neurons and GIs, which lost the connection with the right sensory neurons. It was previously shown that forming new synaptic connections between afferent neurons and collateral interneurons may be the reason for the improved ability to recognize the direction of stimuli after ear elimination in crickets^39,40^.

The mechanisms underlying the EMF effects are not well-known. Cuccurazzu et al.^41^ demonstrated that EMF exposure (50 Hz, 1 mT, 1–7 h/day for 7 days) significantly enhanced neurogenesis in the dentate gyrus of adult mice. 30 days after EMF treatment, the newly generated hippocampal neurons were integrated into the pre-existing network, thereby increasing hippocampal synaptic plasticity^41^. We have found that 3 weeks after the insult, the R/L ratio was much higher for insects exposed to EMF than for non-exposed insects. That may indicate that EMF exposure can effectively drive plasticity processes in the neuronal pathway. A very similar pattern of response was found after the application of the neurotrophic factor TGF-β. The TGF-β-induced improvement of the plasticity of the cockroach escape system was confirmed at the CON level.

Changes in motor activity in insects exposed to EMF were also observed in our study. Three weeks after the injury, the motor activity in EMF-exposed animals was higher than in the non-exposed insects. In previous experiments, we demonstrated that exposure to the EMF of the same value caused a significant increase in cockroach motor activity^42^. The increase in locomotion expressed as travel distance, time in movement, and average speed while in motion after exposure to EMF (50 Hz, 10 mT) was reported for the cockroach also by Todorović et al.^43^. This increase corresponds to the increase in LCN activity after LC stimulation. Insects from the I-Ctr group, 24 h after the injury, were more active than before the *cercus* ablation. The stress induced by the injury might be responsible for an increase in octopamine release, the stress hormone in insects. It is known that octopamine evokes and increases walking movements in cockroaches and other insects, and that octopamine level increases concomitantly with the increase of neuronal activity^44,45^. Moreover, the beneficial impact of octopamine on synapses formation was proved^46^. The results related to the EMF effects on the neuroendocrine system of insects are sparse. It was shown, i.e., that octopamine may participate in the increase in cockroach behavioral activity induced by EMF exposure^42^. Also, another study showed that the static electric field exposure elevated biogenic amine levels, including octopamine in the *Drosophila* brain^47^. The increase in motor activity after EMF exposure observed in the present study can also be associated with the octopamine level increase.

In the presented study, for the first time, we have proved the existence of compensatory plasticity in *Periplaneta americana*. Moreover, using electrophysiological and behavioral studies, we have shown the beneficial impact of EMF on the compensatory neuroplasticity processes.

Particularly:

1. Compensatory plasticity in the cockroach escape system allows the takeover of the function of one *cercus* by the second one.
2. The plasticity processes are improved by the application of TGF-β and exposure to EMF.
3. The results of the presented study exposed that the escape system of *P. americana* can be a recommended model for studying the non-synaptic mechanisms of plasticity, useful for neurorehabilitation research.
4. Considering the positive effect of EMF in post-injury compensation, we recommend further research on EMF in nerve-injury therapies.

## Methods

### Material

Experiments were carried out on adult male cockroaches *(Periplaneta americana)* from the lab-grown colony. The animals were kept at 29 ± 2 °C in the dark and fed with oat flakes and dog chow. Before the experiment (24 h), the insects were moved to 25 °C, the temperature at which the procedures were carried out. The insects were divided into 4 groups: (1) NI-Ctr: not-injured control, (2) I-Ctr: injured control, (3) I-EMF: injured, exposed to EMF, (4) I-TGF: injured, treated with TGF-β. In animals from the injured groups (I-Ctr, I-EMF, I-TGF), the right *cercus* (RC) was carefully ablated on the first day of experiment.

### Procedures

**Figure 5:**
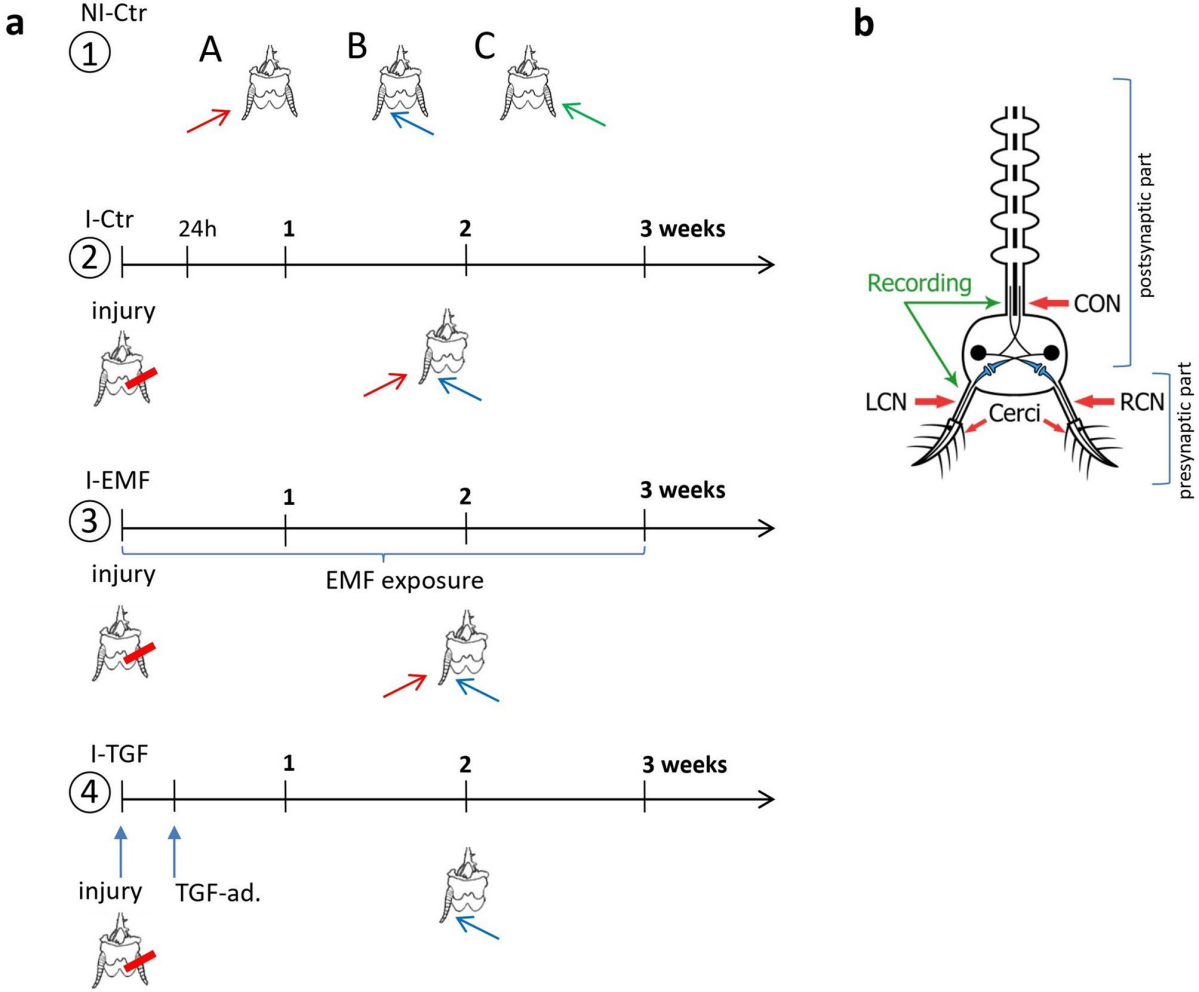
(**a**) Sets of experiments. (1) Initially control nerve activity was recorded in insects from non-injured group (NI-Ctr). Recordings were performed during stimulation of the LC from the left (A) and the right (B) side and stimulation of the RC from the right (C) side. (2) In second set of experiments after RC removal (I-Ctr), nerve activity was recorded during stimulation of LC from the left and right side, in sequence. Measurements were performed 24 h, 1, 2 and 3 weeks after injury. (3) The I-EMF group was exposed to EMF after removal of RC, for 3 weeks. Recordings during stimulation of LC from both sides, in sequence, were collected after 1, 2 and 3 weeks. (4) In the last step of experiments TGF-β was administered to animals after *cercus* removal. Recordings during stimulation of LC from the right side were collected 1, 2 and 3 weeks after injury and TGF-β treatment. (**b**) Schematic organization of neurons constituting the escape system of *P. americana*. The sensory part begins at the *cerci* appendix, where the sensory neuron cell bodies can be found. Left cercal nerve (LCN) and right cercal nerve (RCN) consist of axons of sensory neurons from left *cercus* (LC) and right *cercus* (RC), accordingly. In the terminal abdominal ganglion, sensory neurons form connection with giant interneurons, which axons form the connective nerve (CON) leaving the ganglion. The places for the recording of the bioelectrical activity are marked. The figure was created using Adobe Photoshop CS3 (https://www.adobe.com).

The first set of experiments was performed on the NI-Ctr insects to evaluate pre-injury control values of the parameters (Fig. 5a). All subsequent series of experiments were carried out on insects without RC. In the second set of experiments, the left *cercus* (LC) of the I-Ctr insects was stimulated from both sides, particularly the effects of stimulation from the right side were analyzed. It was assumed that over 3 weeks, the ability to better recognize and process the information coming from the stimulation at the right side will increase for cockroaches with only LC. In the third set of experiments, performed on the I-EMF group, the effect of electromagnetic field (EMF) exposure on the progress of the LC recognition of right-sided stimulation was evaluated. The last set of experiments was performed on the TGF-β-treated group (I-TGF), which served as a positive control.

### Chemicals

Physiological saline for the electrophysiological experiments was prepared with compounds (mM): NaCl—210, KCl—3.1, CaCl_2_—5, MgCl_2_—5.4, Hepes—5; pH = 7.4 was adjusted with NaOH (chemicals from POCH. SA. Poland). Human TGF-β1 (ProSpec-Tany TechnoGene Ltd., New-York, USA) was dissolved in ethanol at the concentration of 10 mg/mL and then diluted with physiological saline to the final concentration of 100 ng/mL.

### EMF exposure

The I-EMF group of cockroaches was exposed to EMF (50 Hz, 7 mT) for 1 h, daily, between 9.00 and 10.00 am, starting the day after the RC removal. The exposure was performed in a 20 cm diameter coil (Elektronika i Elektromedycyna Sp. J.; Poland) and lasted from 1 to 3 weeks. A plastic chamber (11 cm × 16 cm × 22 cm) with insects was placed inside the coil. The I-Ctr group was kept under the same experimental conditions, except for the EMF exposure. Detailed characteristics of the EMF exposure were described by Bieńkowski and Wyszkowska^48^ and Trawiński et al.^49^. The EMF value was measured using a Gauss meter (Model GM2, AlphaLab, Inc, USA). During the experiments, the temperature was monitored in the chamber using thermocouples to ensure to be constant (24.5 ± 1 °C) for all groups.

### TGF-β treatment

Animals (I-TGF group) were administrated with TGF-β one day after the RC removal. A volume of 5 µL TGF-β solution (100 ng/mL) was applied to the insect thorax by Hamilton syringe.

### Electrophysiological recordings

The influence of EMF exposure on the nervous system plasticity was tested in vitro on the abdominal part of the cockroach escape system. The experimental setup for extracellular recordings of the bioelectrical activity of the ventral nerve cord was used^50^. Briefly*, cerci* (or *cercus*) together with the cercal nerves and the abdominal nerve cord (see Fig. 5b) were isolated from the body of the cockroach. The preparation was placed in a 3.5 cm Petri dish and slowly perfused with physiological saline. *Cerci* were kept dry. Electrophysiological recordings were performed using a modified professional extracellular electrode (Alpha Omega Engineering LTD, Israel) from two nerves: the presynaptic cercal nerve and the postsynaptic connective nerve leaving the terminal abdominal ganglion (TAG) (Fig. 5b). A reference non-polarized electrode was placed in the vicinity of the TAG. The electrodes were connected by a preamplifier with a differential amplifier. Bioelectrical signals were displayed on an oscilloscope Hameg 507 (Hameg Instruments, Germany), stored and analyzed using modified Hameg software (version 6.0, Toruń, Poland).

LC or RC was stimulated by an air puff, evoked by a loudspeaker membrane movement (directed by a 0.2 cm tube), controlled by a generator, with 0.4 Hz frequency. Under such stimulation, the increase in nerves activity (response to stimulation) was observed in both cercal and connective nerves. The air puffs were applied on LC first from the left side, then from the right side; the bioelectrical signals were collected first from the cercal nerve and then from the connective nerve. Stimulation of the right cercal nerve was performed by setting the stimulator on the right side of the *cercus* scar. The magnitude of the nerve activity was calculated as before—the duration of the response was multiplied by the amplitude of the signals at each point of recording^50^.

### In-vivo behavioral tests

The influence of EMF exposure on the behavior of cockroaches was tested in vivo using a locomotor test. The cercal appendix is responsible for the detection of air movement around the insects, thus it is necessary to proper spatial orientation and “recognition of environment”. We have observed that the ablation of one *cercus* changes the behavior of cockroaches. Consequently, we accepted the changes in motor activity as a marker of changes in the ability of proper stimuli perception. Five insects were placed in a 50-cm diameter glass arena. The insect movements were recorded using a video-camera (Logitech 1080) for 10 min. Video files were processed with the idTracker software (Stoelting, CO, USA)^51^, and the output files were then analyzed using *ad-hoc* scripts developed with MATLAB (version R2020a, The MathWorks, Inc., Natick, Massachusetts, USA, https://www.mathworks.com).

### Statistical analysis

The analyses were made using ANOVA or Kruskal–Wallis tests for few data with non-normal distribution. Differences in group sizes were not significant and the groups were homogenous. The differences between groups were tested by Gabriel *post-hoc* test or Mann–Whitney test. All analyses were conducted in the IBM SPSS 25 Statistics software (IBM Corporation, Armonk, NY, USA). The results were expressed as mean values ± SE. The differences were considered significant when p < 0.05.
